# Comparative transcriptomics reveals unique pine wood decay strategies in the *Sparassis latifolia*

**DOI:** 10.1038/s41598-022-24171-z

**Published:** 2022-11-18

**Authors:** Chi Yang, Lu Ma, Donglai Xiao, Xiaoyu Liu, Xiaoling Jiang, Yanquan Lin

**Affiliations:** 1 Institute of Edible Mushroom, Fujian Academy of Agricultural Sciences, Fuzhou, 350014 China; 2 National and Local Joint Engineering Research Center for Breeding and Cultivation of Featured Edible Mushroom, Fujian Academy of Agricultural Sciences, Fuzhou, 350014 China

**Keywords:** Cell biology, Microbiology, Molecular biology

## Abstract

*Sparassis latifolia* is a valuable edible mushroom, growing on fresh pine wood sawdust substrate. However, the mechanistic bases are poorly understood. The gene expression profiles of *S. latifolia* were analyzed from submerged cultures with fresh pine wood sawdust substrate for different time (0 h, 1 h, 6 h, 1 day, 5 days, and 10 days, respectively). The total number of differentially expressed genes (DEGs) identified under pine sawdust inducing was 2,659 compared to 0 h (CK). And 1,073, 520, 385, 424, and 257 DEGs were identified at the five time points, respectively. There were 34 genes in common at all inoculated time points, including FAD/NAD(P)-binding domain-containing protein, glucose methanol choline (GMC) oxidoreductase, flavin-containing monooxygenase, and taurine catabolism dioxygenase. Weighted gene co-expression analysis (WGCNA) was then used to compare the molecular characteristics among the groups and identified that the blue module had the highest correlation with the time induced by pine wood sawdust. There were 102 DEGs out of 125 genes in the blue model, which were most enriched in nitronate monooxygenase activity, dioxygenase activity, and oxidation–reduction process GO terms (p < 0.05), and peroxisome in KEGG pathway. This may provide clues into mechanisms that *S. latifolia* can grow on fresh pine wood sawdust substrate.

## Introduction

Biodegradation of woody biomass is largely dependent on basidiomycetous fungi^1,2^. One major degraders of forest biomass is brown rot fungi^2^. Wood is degraded by brown-rot fungi using both enzymatic and non-enzymatic processes^3^.

It is generally believed that wood-decaying fungi have substrate specificity that defines their ecological niche^4^. The mechanisms by which fungi grow on a specific substrate are crucial to understanding both the ecology of fungi and the industrial applications of different feedstocks, such as cultivation of edible mushrooms. Pine trees are a common coniferous group of trees that have a significant ecological and economic significance throughout the world. Sawdust from pine trees, however, is not commonly used since most fungi cannot thrive directly on coniferous wood because of the rosin present in pine trees^5^.

*Sparassis latifolia* is a brown-rot fungus that grows primarily on the stumps or roots of coniferous trees^6,7^, which is the commonly cultivated *Sparassis* species in China^8^. A previous study^9^ investigated the potential of producing liquid spawn of *S. latifolia* by submerged fermentation under controlled conditions. It evaluated its ability to colonize on pine sawdust substrate. It was discovered that the transcript levels of genes encoding glycoside hydrolases were mainly governed by carbon source type, while genes involved in hemicellulose degradation were mostly up-regulated^10^. However, the mechanism of pine wood decay by *S. latifolia* is still unclear.

In this study, we examined the gene expression of *S. latifolia* during pine wood decay processing. The sterilized pine sawdust was added into fresh broth cultured mycelia and cultured for 0 h, 1 h, 6 h, 1 day, 5 days, and 10 days, respectively. We found that many of the genes involved in known or predicted functions in wood decay were differentiated expressed, with 34 genes (including 17 up-regulated and 17 down-regulated genes) in common at all inoculated time points. Weighted gene co-expression analysis (WGCNA) identified that the blue module had the highest correlation with the time induced by pine wood sawdust, in which 102 DEGs out of 125 genes were most enriched in nitronate monooxygenase activity, dioxygenase activity, and oxidation–reduction process GO (gene ontology) terms, and peroxisome in KEGG pathway.

## Results

### RNA sequencing and mapping

To investigate the profile of gene expression during pine sawdust inducing (PSI), samples with three biological replicates were submitted for RNA-Seq. We totally obtained 71.91 Gb clean data after RNA sequencing for 18 samples with at least 3.38 Gb clean data for each sample. And in each sample, more than 93.84% of bases score Q30 and above (Table 1). The mapping ratio varying from 94.38% to 95.03% (Table 2).

**Table 1:** Sequencing data Statistics.

Samples	Clean reads	Clean bases	GC content (%)	% ≥ Q30 (%)
CK-1	15,300,218	4,575,935,026	56.47	95.23
CK-2	11,582,063	3,464,275,068	56.38	95.29
CK-3	12,389,483	3,705,763,772	56.33	95.24
T-10d-1	12,786,507	3,815,880,670	56.59	94.07
T-10d-2	13,372,452	3,993,664,028	56.52	93.96
T-10d-3	13,205,631	3,943,933,782	56.55	94.04
T-1d-1	12,475,495	3,730,823,260	56.41	94.19
T-1d-2	12,285,065	3,666,475,380	56.44	93.84
T-1d-3	13,389,505	4,004,268,706	56.53	94.83
T-1h-1	14,400,608	4,302,919,064	56.30	94.55
T-1h-2	15,632,077	4,669,081,164	56.45	94.30
T-1h-3	15,326,678	4,578,054,414	56.56	94.28
T-5d-1	14,517,067	4,339,936,502	56.51	95.12
T-5d-2	11,292,773	3,375,518,776	56.53	95.09
T-5d-3	13,043,860	3,895,294,712	56.42	94.40
T-6 h-1	11,619,691	3,476,037,790	56.54	95.09
T-6 h-2	13,200,926	3,947,577,998	56.44	95.32
T-6 h-3	14,818,151	4,427,615,962	56.52	95.17

**Table 2:** Alignment results of each sample.

ID	Total reads	Mapped reads	Uniq mapped reads	Multiple map reads	Reads map to ' + '	Reads map to '−'
CK-1	30,600,436	29,044,147 (94.91%)	27,956,114 (91.36%)	1,088,033 (3.56%)	14,309,892 (46.76%)	14,390,683 (47.03%)
CK-2	23,164,126	21,999,812 (94.97%)	21,170,957 (91.40%)	828,855 (3.58%)	10,831,411 (46.76%)	10,893,389 (47.03%)
CK-3	24,778,966	23,512,915 (94.89%)	22,564,089 (91.06%)	948,826 (3.83%)	11,552,879 (46.62%)	11,635,737 (46.96%)
T-10d-1	25,573,014	24,203,888 (94.65%)	23,253,077 (90.93%)	950,811 (3.72%)	11,908,815 (46.57%)	11,994,367 (46.90%)
T-10d-2	26,744,904	25,242,211 (94.38%)	23,871,260 (89.26%)	1,370,951 (5.13%)	12,287,318 (45.94%)	12,474,309 (46.64%)
T-10d-3	26,411,262	24,982,441 (94.59%)	23,997,669 (90.86%)	984,772 (3.73%)	12,291,848 (46.54%)	12,380,030 (46.87%)
T-1d-1	24,950,990	23,583,112 (94.52%)	22,689,140 (90.93%)	893,972 (3.58%)	11,611,831 (46.54%)	11,681,768 (46.82%)
T-1d-2	24,570,130	23,212,566 (94.47%)	22,354,731 (90.98%)	857,835 (3.49%)	11,429,652 (46.52%)	11,502,280 (46.81%)
T-1d-3	26,779,010	25,375,908 (94.76%)	24,394,245 (91.09%)	981,663 (3.67%)	12,491,402 (46.65%)	12,574,385 (46.96%)
T-1h-1	28,801,216	27,239,764 (94.58%)	25,754,943 (89.42%)	1,484,821 (5.16%)	13,238,036 (45.96%)	13,428,258 (46.62%)
T-1h-2	31,264,154	29,588,505 (94.64%)	28,423,188 (90.91%)	1,165,317 (3.73%)	14,541,255 (46.51%)	14,632,378 (46.80%)
T-1h-3	30,653,356	28,986,961 (94.56%)	27,913,821 (91.06%)	1,073,140 (3.50%)	14,273,055 (46.56%)	14,350,090 (46.81%)
T-5d-1	29,034,134	27,550,568 (94.89%)	26,475,479 (91.19%)	1,075,089 (3.70%)	13,556,480 (46.69%)	13,649,129 (47.01%)
T-5d-2	22,585,546	21,424,342 (94.86%)	20,566,413 (91.06%)	857,929 (3.80%)	10,536,653 (46.65%)	10,615,840 (47.00%)
T-5d-3	26,087,720	24,674,712 (94.58%)	23,606,986 (90.49%)	1,067,726 (4.09%)	12,106,789 (46.41%)	12,212,529 (46.81%)
T-6 h-1	23,239,382	22,061,603 (94.93%)	21,251,399 (91.45%)	810,204 (3.49%)	10,870,912 (46.78%)	10,925,195 (47.01%)
T-6 h-2	26,401,852	25,089,074 (95.03%)	24,158,588 (91.50%)	930,486 (3.52%)	12,362,925 (46.83%)	12,421,200 (47.05%)
T-6 h-3	29,636,302	28,121,984 (94.89%)	27,061,825 (91.31%)	1,060,159 (3.58%)	13,845,390 (46.72%)	13,924,141 (46.98%)

### Differential gene expression and functional enrichment analysis

**Figure 1:**
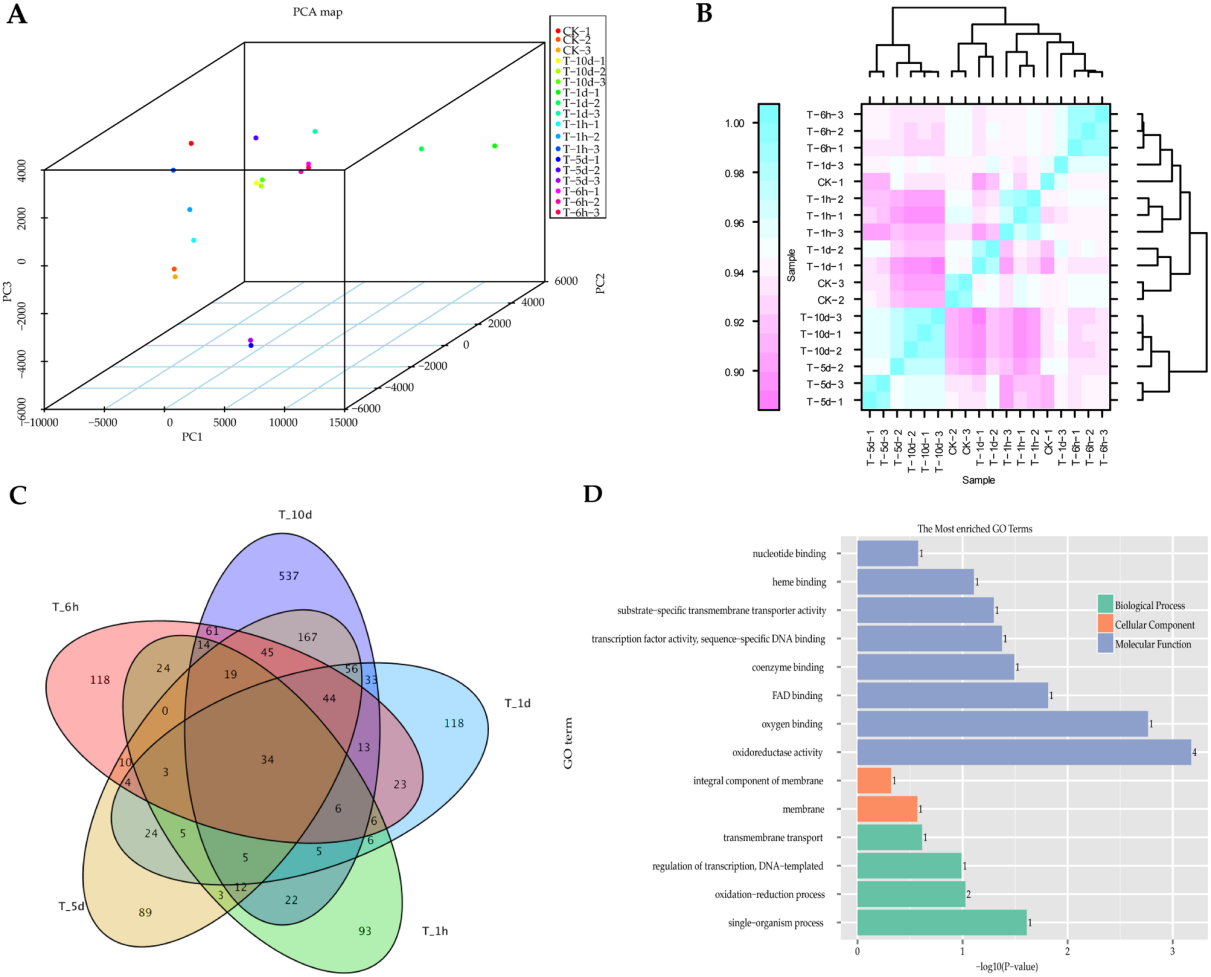
The differentially expressed genes (DEGs) in PSI. (**A**) PCA plot. (**B**) Correlation heat map performed using BMKCloud (http://www.biocloud.net). (**C**) The venn diagram for the DEGs shared between the five time points.

To further confirm the quality of RNA-seq, PCA and pearson correlation analysis were performed. Based on the correlation results for samples in Fig. 1A,B, CK-1, T-1h-3, T-1d-3, and T-5d-2 were excluded for further analysis. During identifying differentially expressed genes (DEGs), |FoldChange|> 2.0 and adjusted P-value < 0.01 were used as the screening criteria. In total, 2,659 DEGs were identified under pine sawdust inducing; we identified 1073, 520, 385, 424, and 257 DEGs at the five time points, respectively (Table 3). There were 34 genes in common at all inoculated time points, including 17 up-regulated and 17 down-regulated genes (Fig. 1C and Table S1). These 34 genes were significantly enriched in single-organism process biological processes (BP) and oxidoreductase activity, oxygen binding, FAD binding, coenzyme binding, and transcription factor activity, sequence-specific DNA binding cellular component (CC) (p < 0.05) (Fig. 1D, Table S2). However, there were no significantly enriched KEGG pathways for these 34 genes.

**Table 3:** The number of DEGs at the five time points compared to control group.

Groups	DEGs_total	DEGs_up	DEGs_down
1 h	257	154	103
6 h	424	294	130
1 d	385	176	209
5 d	520	306	214
10 d	1073	481	592

### Weighted correlation network analysis

**Figure 2:**
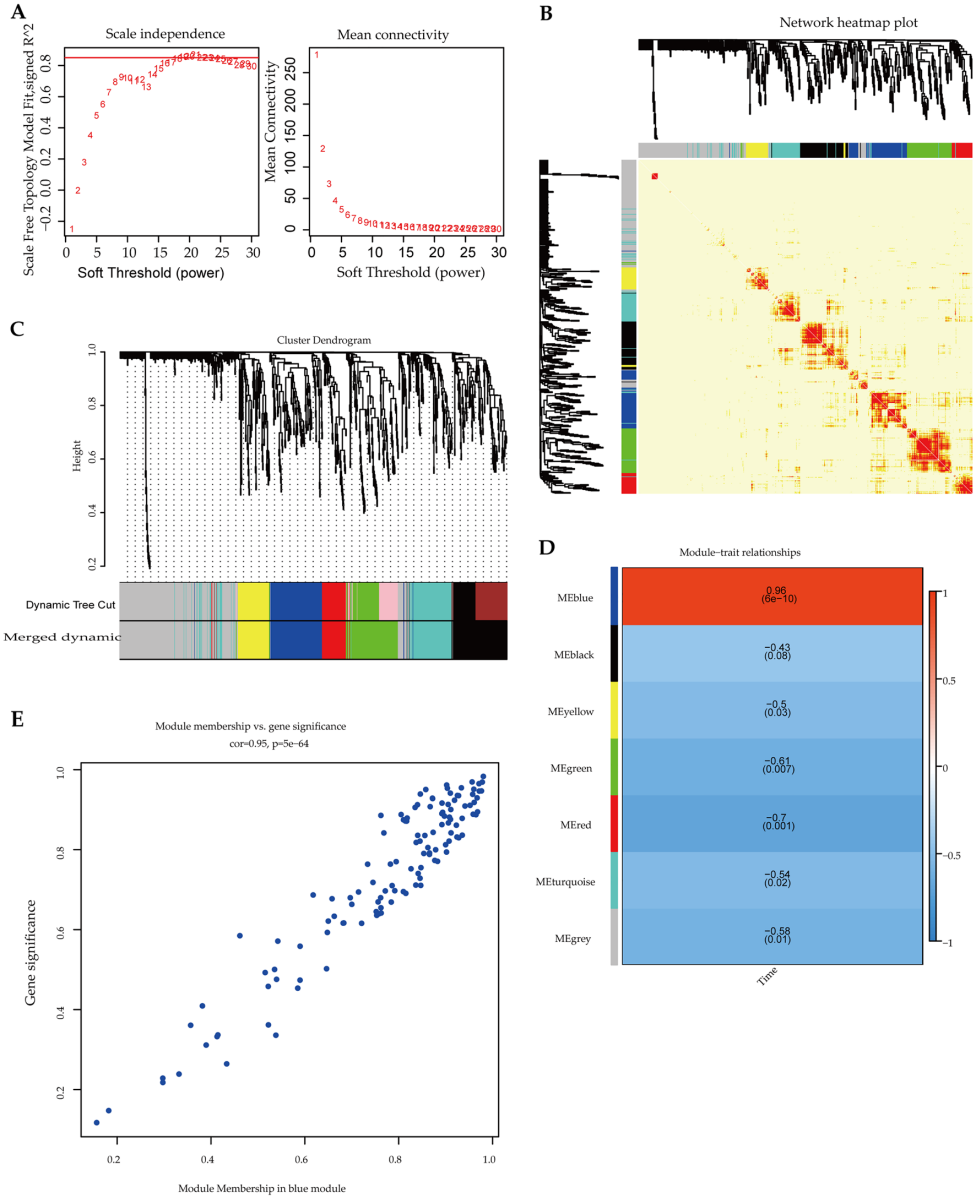
Construction of WGCNA analysis using BMKCloud (www.biocloud.net). (**A**) The soft-threshold power versus scale-free topology model fit index and mean connectivity. The left image shows the scale-free fit index (y-axis) as a function of the soft-thresholding power (x-axis). The right image shows the average connectivity (degree, y-axis) as a function of the soft-thresholding power (x-axis). (**B**) Heat map of the correlation between modules and traits. (**C**) Module clustering tree. (**D**) Heatmap shows correlations of module-related genes and the treatment time of pine sawdust. (**E**) A scatter plot of gene significance (GS) vs. module membership (MM) in the blue module.

Through correlation analysis, the gene modules related to specific sample traits could quickly screened from the data. Specifically, we identified seven functional modules related to PSI by WGCNA. As a result, clustering analysis was carried out on gene expression data from 18 libraries using the average linkage method and pearson’s correlation method. The soft threshold power value of β = 19 was selected to obtain a scale-free co-expression network (Fig. 2A). Seven modules were identified based on average hierarchical clustering and dynamic tree clipping (Fig. 2B,C). As shown in Fig. 2D, the blue module had the highest correlation with the time of PSI (cor = 0.96) and was therefore selected for subsequent analysis. After performing the gene significance against module membership, we observed that genes with high module memberships tended to have high gene significance in the blue module (cor = 0.95, *P* = 5e−64; Fig. 2E).

### Function annotation of DEGs in blue module

**Figure 3:**
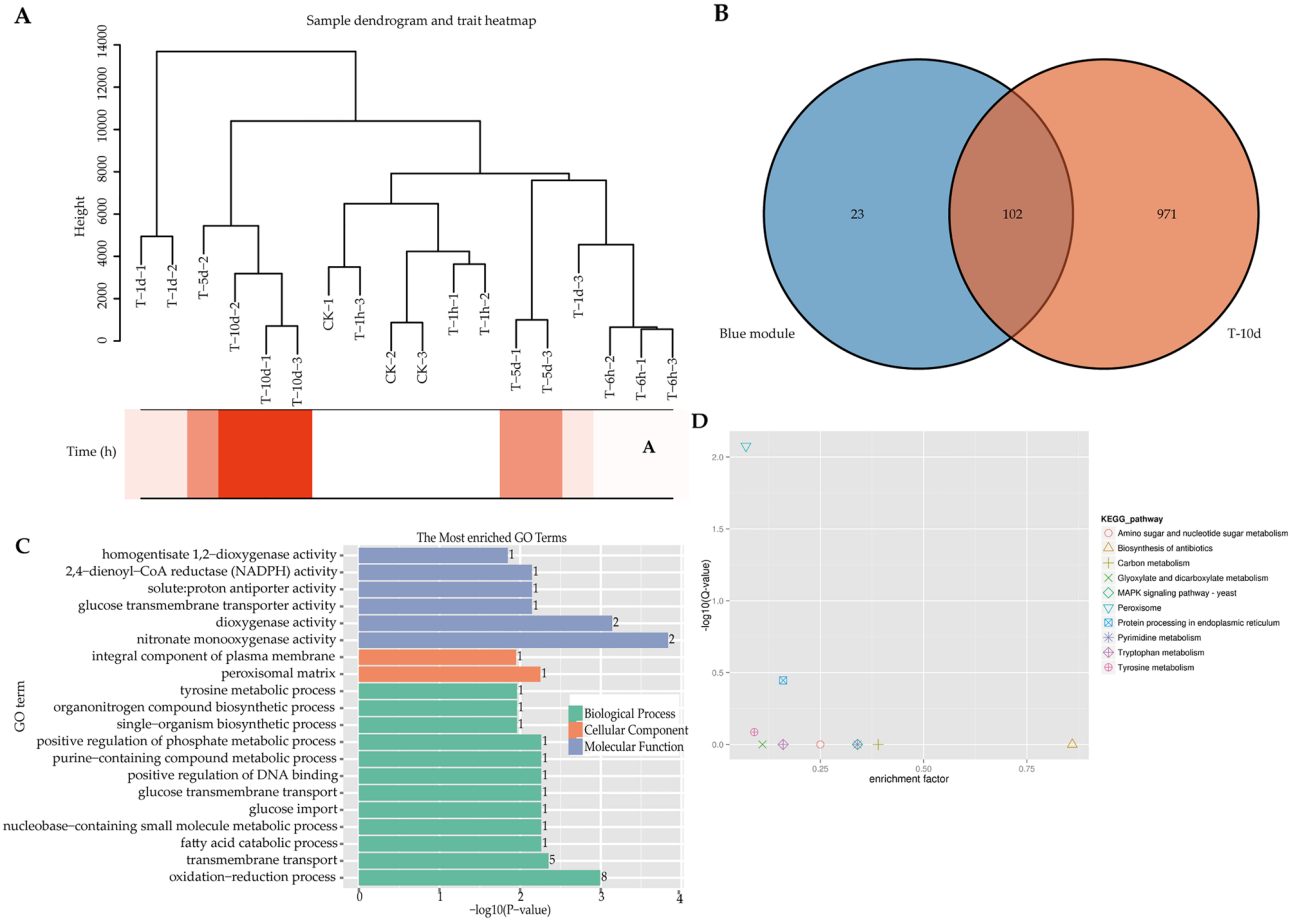
Function annotation of DEGs in blue module. (**A**) Sample dendrogram and trait heatmap. (**B**) The venn diagram for the blue module and DEGs in T-10d vs control group. (**C**) GO functional enrichment analysis. (**D**) KEGG pathway enrichment analysis.

There were 125 genes in the blue module. In order to screen out the core genes that are more relevant and highly correlated to the module eigengene, we then checked the gene expression in T-10d group, which was significantly correlated with the time of PSI (Fig. 3A). Compared to control group, there were 102 DEGs screened out (Fig. 3B, Table S3). In order to systemically investigate the function and pathway of the blue module, we further performed GO and KEGG pathway enrichment analysis. According to the annotation results (Fig. 3C,D), the DEGs in blue module were most enriched in nitronate monooxygenase activity, dioxygenase activity, and oxidation–reduction process GO terms, and peroxisome in KEGG pathway (p < 0.05).

### Expression validation of hub genes

**Figure 4:**
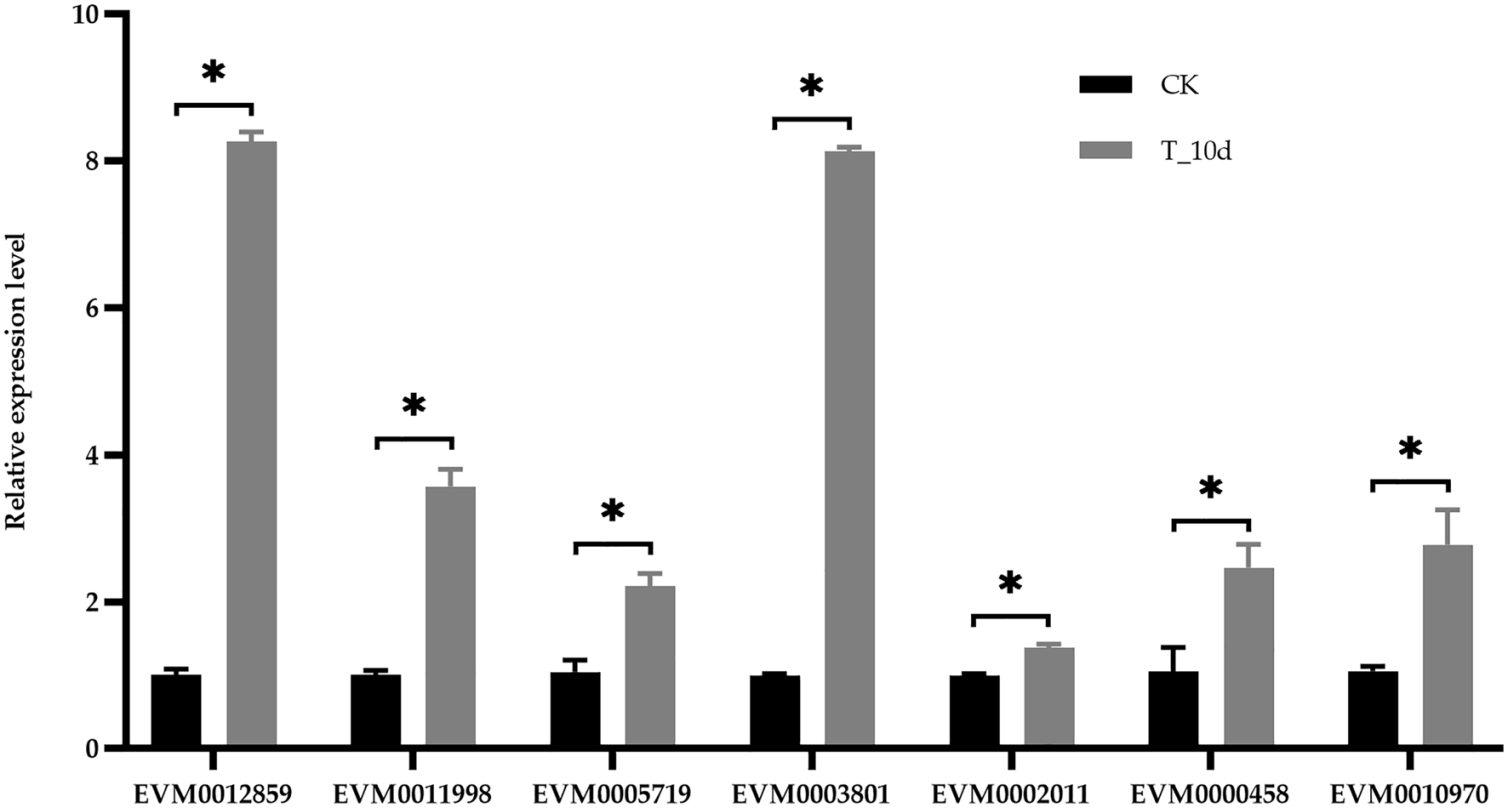
Expression validation of selected genes by qRT-PCR.

Based on the results of function annotation, five genes were selected for further validation, including EVM0002011, EVM0005719, and EVM0011998 in peroxisome (ko04146) KEGG pathway, and EVM0003801 and EVM0012859 in nitronate monooxygenase activity (GO:0018580) GO term (Table S4). Meanwhile, EVM0010970 and EVM0000458 were also selected because of up-regulating in all comparison and previous studies^3,4^, respectively. The module membership of selected genes was ranged from 0.7780 to 0.9777 (Table S5). qRT-PCR was used to quantitatively validate the sequencing data. GAPDH was selected as the reference gene because of its stable expression level based on our previous study^11^. Melt curve analysis of all genes showed no primer dimers or nonspecific product amplification (Fig. S1). As shown in Fig. 4, all the seven genes had the similar expression pattern to the RNA-seq results.

## Discussion

Mushroom-forming fungi are important wood-degraders in global carbon cycling. However, wood-decaying fungi tend to have characteristic substrate ranges. Most wood-decaying fungi are unable to colonize coniferous wood directly due to the rosin resent in pine trees^5^. There were only little studies that focus on the pine wood decay, the fungi included *Fomitopsis pinicola*^4,12^, *Antrodia sinuosa*^12^, *Postia placenta*^12^, *Wolfiporia cocos*^12^, *Laetiporus sulphureus*^12^, *Daedalea quercina*^12^, *Rhodonia placenta*^13^ and *Schizophyllaceae*^14^. However, all these studies were not indicated that the pine wood used was fresh.

Sawdust from fresh pine trees is not commonly utilized and most fungi cannot colonize coniferous wood directly^5^. However, our previous study revealed that *S. latifolia* can grow on fresh pine wood sawdust substrate^9,10^. So, we hypothesized that there were some special genes in *S. latifolia* to ensure it could grow on fresh pine wood. In our results, the 34 common DEGs in all time points were searched out (Fig. 1C, Table S1). Some of these DEGs were reported in previous studies, including GMC oxidoreductase (EVM0000788)^12^, FAD/NAD(P)-binding domain-containing protein (EVM0008017)^4^, Flavin-containing monooxygenase (EVM0013062)^3^, and Taurine catabolism dioxygenase (EVM0008301)^3^. However, most DEGs were function unknown or had no related function report.

Based on the results of WGCNA, the blue module had the highest correlation with the 10 h of PSI (Fig. 2D). 102 out of 125 genes in the blue model were significantly differentiated expressed under 10 h PSI (Table S2). Among these genes, short-chain dehydrogenase (EVM0010558, EVM0003708, EVM0005719, EVM0000652, EVM0006859)^3^, glycoside hydrolase family proteins (EVM0000458)^3,4^, and lipase (EVM0003260, EVM0000539)^3,4^ were wood decay related. However, there were only one common gene (EVM0010970) with the 34 common DEGs in all treatment groups compared to control.

Sawdust from fresh pine trees usually cannot directly use to cultivate edible mushrooms and the reasons remain unclear to now. A previous study showed that rosin is an abundant raw material from pine trees, resulting in most fungi are unable to colonize coniferous wood directly^5^. Rosin is a solid and brittle mixture of non-volatile conifer tree resin components. Many studies^15–17^ have done to revealed the compositions of rosin. Resin acids, with the general formula C_19_H_29_COOH, constitute up to 95 wt.% of rosin^18^. Components from pinus species had been shown to have antimicrobial activity^19,20^. However, it is unclear which component from fresh pine wood inhibit the growth of edible mushroom. This remains to be further investigated.

Although most edible mushroom cannot grow well on fresh pine sawdust, several species can directly utilize, such as *Fomitopsis pinicola*^3,4^, *Wolfiporia cocos*^21^, and *S. latifolia*^9^. In the future study, we can compare the difference between these two kinds of species to explore the mechanism of fresh pine wood decay by combining genomics, transcriptomics, proteomics, metabolomics and epigenomic.

## Conclusions

The gene expression profile of *S. latifolia* under pine sawdust inducing were obtained and key genes were screened by WGCNA. This maybe provides clues into mechanisms that *S. latifolia* can grow on fresh pine wood sawdust substrate.

## Materials and methods

### Strain and sample preparation

The *S. latifolia* strain SP-C was preserved at the Institute of Edible Mushroom, Fujian Academy of Agricultural Sciences (Fuzhou, China). The strain was cultured as previous study^9^ with modification. Potato dextrose agar (PDA) slants were used to grow the strain, and the seed culture medium contained potato (20%), glucose (2%), and fish peptone (0.3%). The mycelia were activated for ten days at 25 °C in darkness on a PDA slant. Mycelial plugs (2 mm in diameter) were cultured with 100 mL of liquid media in 250 mL flasks in a rotatory incubator at 25 °C with shaking at 150 rpm/min for 9 days in darkness. And then, treating of pine wood sawdust, culturing, and harvesting of mycelium was conducted as prior study^12^ with modification. After homogenizing, the spawn was transferred to a new 250 mL flasks containing 100 mL of liquid media and cultured for 2 days. Then, 1 g sterilized fresh pine sawdust was added at different time, so that the pine sawdust inducing time were 0 h, 1 h, 6 h, 1 days, 5 days, and 10 days, respectively. Finally, the strains were collected at the same time by filtering to remove sawdust.

### RNA sequencing

Total RNA was isolated by using TRIzol (Invitrogen, USA) according to the manufacturer’s instructions. Agilent 2100 Bioanalyzeror SMA3000 was used to measure RNA purity, concentration, and RNA integrity number. RNA-Seq was performed as previously described^22^. Briefly, From total RNA, mRNA was enriched using poly (T) + oligo beads, eluted with Tris–HCl buffer, then fragmented using RNA fragmentation kits (Ambion, Austin, TX, USA). A random hexamer primer and M-MuLV Reverse Transcriptase were used to make first strand cDNA. Subsequently, second strand cDNA synthesis was carried out using DNA Polymerase I and RNase H. By using exonuclease/polymerase, the remaining overhangs were converted into blunt ends. In order to prepare for hybridization, NEBNext Adaptors with hairpin loop structure were ligated after 3'-adenylation of DNA fragments. A purification procedure was performed using AMPure XP (Beckman Coulter, Beverly, USA) to select cDNA fragments of 240 bp or less in length from the library fragments. With size-selected, adaptor-ligated cDNA, 3 ul USER Enzyme (NEB, USA) was used at 37 °C for 15 min and then at 95 °C for 5 min prior to PCR. Next, PCR was performed using Phusion DNA polymerase, Universal PCR primers, and Index (X) primer. Final steps included purifying PCR products (AMPure XP system) and assessing the quality of the libraries (Agilent Bioanalyzer 2100 system). In accordance with the manufacturer's instructions, the indexed samples were clustered using TruSeq PE Cluster Kit v4-cBot-HS (Illumia) on a cBot Cluster Generation System. Using an Illumina nova 6000 platform with the PE150 approach were generated from the library preparations after cluster generation.

### Read mapping, annotation, and quantification of gene expression

The raw paired-end reads were checked using the FastaQC Package (http://www.bioinformatics.babraham.ac.uk/projects/fastqc/). By using Cutadapt, adaptor sequences, low-quality sequence reads, and the short reads (< 20 bp) were removed from the data sets^23^. Clean reads were generated after raw sequences were processed. These clean reads were mapped to the reference genome sequence of *S. latifolia*^24^ by Hisat2 tools (version 2.0.4)^25^. Reads were aligned to the reference genome and then assembled into transcripts by StringTie (version 1.3.3b) using default parameters (version 2.0.6)^26^. Transdecoder (version 2.0.1) were used to predict coding sequences. Gene function was annotated based on seven databases, including Nr (NCBI non-redundant protein sequences), Nt (NCBI non-redundant nucleotide sequences), Pfam (Protein family), KOG/COG (Clusters of Orthologous Groups of proteins), Swiss-Prot (A manually annotated and reviewed protein sequence database), KO (KEGG Ortholog database), and GO (Gene Ontology). Then, picard-tools v1.41 and samtools v0.1.18 were used to sort, remove duplicated reads and merge the bam alignment results of each sample. Gene expression patterns were quantified using STAR-RSEM algorithm (version 4.1). The mapped read numbers were calculated and normalized by RESM-based algorithm in the Trinity package^27^.

### Differential expression analysis

Principal component analysis (PCA) was utilized to reduce and summarize large datasets while illustrating relationships between samples based on co-variance of the data being examined. The mapped reads of the 500 genes that had the largest coefficients of variation based on the fragments per kilobase of transcripts per million (FPKM)^28^ were used for PCA and heat mapping with unsupervised clustering. Based on read counts, the pair-wise spearman correlation between any pair of samples was calculated. DESeq2 R package was used to analyze the differential expression^29^. The resulting P values were adjusted using the Benjamini and Hochberg’s approach for controlling the false discovery rate. Genes with |FoldChange|> 2.0 and adjusted P-value < 0.01 were assigned as differentially expressed.

### WGCNA analysis

The weighted gene co-expression network analysis (WGCNA) was used to identify the functional modules, which has advantages to find the complex relationships between relating modules and associated with traits^30^. Specifically, the lowest soft-thresholding power for which the scale-free topology fit was selected. The topological overlap matrix (TOM) similarity was calculated using the power and expression data of all genes. The clustering tree structure of the TOM were constructed by hierarchical clustering method. Different branches of the clustering tree represent different gene modules, and different colors represented different modules. Genes with similar patterns were grouped into one module according to the weighted correlation coefficients of genes. The correlation between co-expression modules and traits was estimated based on the phenotypic information of pine wood sawdust inducing time of 0 h, 1 h, 6 h, 1 day, 5 days, and 10 days. A significant co-expression module highly related to traits was identified. Module–trait relationships were computed by Pearson’s correlation tests, and *P* < 0.05 was defined significant correlation. WGCNA analysis was performed using BMKCloud (https://www.biocloud.net). Gene Ontology (GO) enrichment analysis was implemented by the GOseq R packages based Wallenius non-central hyper-geometric distribution^31^. We used KOBAS ^32^ software to test the statistical enrichment of differential expression genes in KEGG pathways^33–35^.

### Gene expression validation by quantitative RT-PCR

Gene expression analysis was performed by qRT-PCR as previously described^8^. Briefly, total RNA was isolated using TRIzol reagent (Invitrogen) and then reverse transcribed with PrimeScript™ II 1st Strand cDNA Synthesis Kit (Takara, Japan) following the manufacturer’s instructions. cDNA was quantified using SYBR Premix Ex Taq kit (Takara, Japan) on an ABI QuantStudio instrument. The GAPDH gene was selected as control^11^. Primers used in this study were described in Supplementary Table S6. The reaction mixture contained 4.5 μL cDNA, 0.5 μL primers (10 μM), 10 μL 2× SYBR Premix Ex Taq, and ddH_2_O up to 20 μl. The thermal cycling conditions were: 95 °C for 1 min; followed by 40 cycles of 10 s at 95 °C, 34 s at 60 °C; and 60 °C for 1 min, and 60 °C to 95 °C for the dissociation curve analyses. Three biological replicates were used. qRT-PCR data were presented as mean ± SD. The relative gene expression was calculated using the 2^−ΔΔCT^ method. GraphPad Prism 8 software was employed for multiple t tests. Statistical significance determined using the Holm-Sidak method, with *P < 0.05.

## Supplementary Information

Supplementary Figure S1.

Supplementary Tables.

## Data Availability

The RNA-Seq data had been deposited in NCBI under accession GSE173822.
