# Supplementary material for: Comparative transcriptomics reveals unique pine wood decay strategies in the Sparassis latifolia
Source: Sci Rep. 2022 Nov 18;12:19875. doi: 10.1038/s41598-022-24171-z (PMC9674834; doi:10.1038/s41598-022-24171-z)
Supplement: Supplementary file 1 — Supplementary Figure S1. [file 41598_2022_24171_MOESM1_ESM.pdf]

Comparative transcriptomics reveals unique pine wood decay strategies in the *Sparassis latifolia*

Chi Yang[1], Lu Ma[1], Donglai Xiao[1], Xiaoyu Liu[1], Xiaoling Jiang[1], Yanquan Lin[1,*]

1 Institute of Edible Mushroom, Fujian Academy of Agricultural Sciences, Fuzhou 350014, China; National and Local Joint Engineering Research Center for Breeding & Cultivation of Featured Edible Mushroom, Fujian Academy of Agricultural Sciences, Fuzhou 350014, China; yc113078@163.com, malujj@163.com，xdljiangsu@163.com, 867119118@qq.com, gone_to@163.com, lyq-406@163.com

* Correspondence: lyq-406@163.com

Supplementary Information

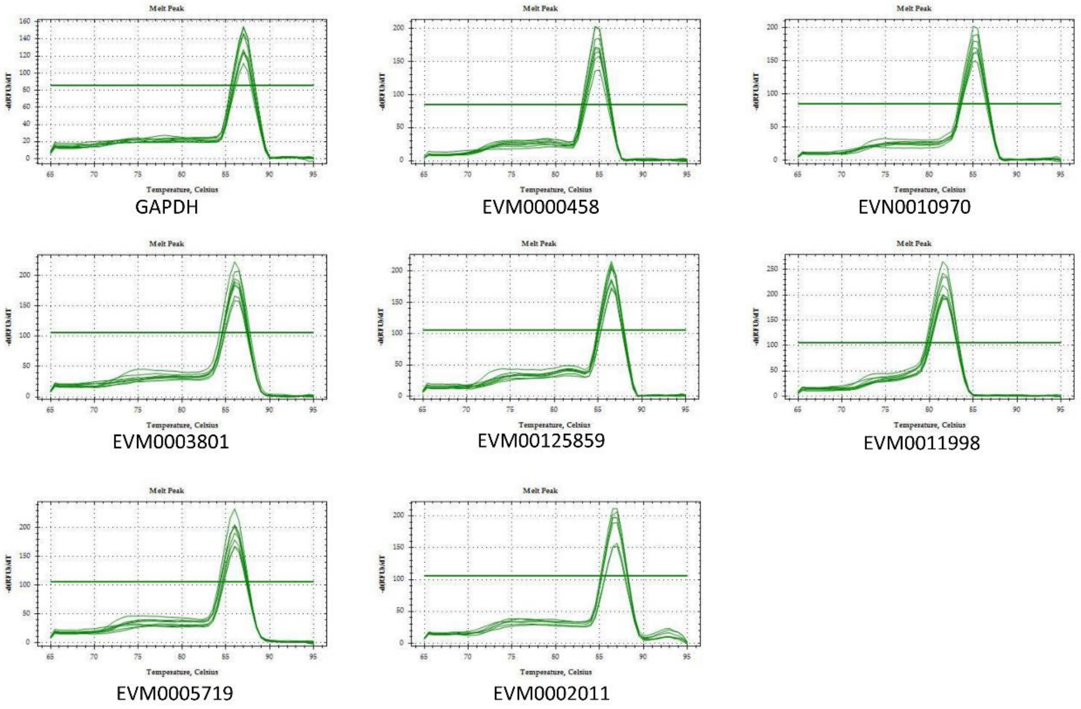

Figure S1. The melt curve of the primers for the selected validation genes
